# Modifying Effects of Glucose and Insulin/Insulin-Like Growth Factors on Colon Cancer Cells

**DOI:** 10.3389/fonc.2021.645732

**Published:** 2021-07-05

**Authors:** Şeyda Berk, Joseph A. M. J. L. Janssen, Peter M. van Koetsveld, Fadime Dogan, Naci Değerli, Servet Özcan, Fahrettin Kelestimur, Leo J. Hofland

**Affiliations:** ^1^ Department of Molecular Biology and Genetics, Faculty of Science, Sivas Cumhuriyet University, Sivas, Turkey; ^2^ Department of Internal Medicine, Division of Endocrinology, Erasmus Medical Center, Rotterdam, Netherlands; ^3^ Genome and Stem Cell Center (GENKOK), Erciyes University, Kayseri, Turkey; ^4^ Department of Biology, Faculty of Sciences, Erciyes University, Kayseri, Turkey; ^5^ Yeditepe University, Faculty of Medicine, Istanbul, Turkey

**Keywords:** colorectal cancer, glucose, insulin, insulin-like growth factors, IGF-IR, IR

## Abstract

There are only a few experimental studies which have investigated effects of glucose alone, and glucose in combination with insulin/insulin-like growth factors (IGF) on the growth of colon cancer. In the present study, we studied *in vitro* in human colorectal cancer cells originating from four Dukes’ stages of colorectal cancer the effects of glucose, insulin and IGFs on proliferation, migration, cell cycle progression and gene expression of the IGF system. Growth of colon cancer cells originating from a Dukes’ stage A was glucose-dependent, whereas growth of cancer cells from Dukes’ stage B, C and D was glucose-independent. Stimulatory effects of insulin and IGFs on cell growth were observed only in colon cancer cells originating from Dukes’ stage C and D. IGF-II stimulated migration in Dukes’ stage B cells only. The growth stimulatory effects in Dukes’ stage C and D colorectal cancer cells were accompanied by G2/M arrest and associated with an increased IGF-IR/IGF-II receptor ratio. In conclusion, our *in vitro* data suggest that the stimulating effects of glucose, IGFs and insulin on proliferation differ between colorectal cancer cells from early and late Dukes’ stages. Stimulatory effects of glucose on proliferation appear predominantly present in stage Dukes’ stage A colorectal cancer cells, while in contrast growth factor-mediated stimulation of cell proliferation is more pronounced in Dukes’ late stage (metastasized) colorectal cancer cells. Moreover, our study suggests that a stringent glucose control may be important to control tumor growth in early stages of colorectal cancer, while inhibition of the endocrine actions of the IGFs and insulin become more important in the late (metastasized) stages of colorectal cancer to restrain growth of colon cancer cells.

## Introduction

Colorectal cancer and type 2 diabetes mellitus are main causes of morbidity and mortality worldwide (1–3). Epidemiologic evidence suggests an association between diabetes mellitus and an increased risk of colorectal cancer (4–7). Several studies have suggested that hyperinsulinemia and elevated glucose levels increase the risk for colorectal cancer (4, 7–15). Hyperinsulinemia may stimulate insulin receptors (IR) and/or insulin-like growth factor receptors (IGFIR/IGFIIR) and thereby induce cell proliferation and suppress apoptosis (16–18).

The IGF signaling pathway has been considered to be involved in the proliferation and differentiation of epithelial cells of the colon, breast, lung and prostate (19). The IGF system is complex and consists of at least two insulin-like growth factor receptors (IGF-IR and IGF-IIR), two polypeptide ligands (IGF-I and IGF-II) and six binding proteins (IGFBP-1-6) (20). In addition, a large group of insulin growth factor binding protein proteases may cleave IGFBPs and increase levels of free IGF-I and IGF-II (21). It is thought that both IGF-I/II and the IGF-IR by inducing cellular proliferation, growth and immunosuppression play important roles in tumorigenesis (22–25). Elevated circulating levels of IGF-I and IGF-II have been associated with an increased risk on colorectal cancer (25, 26). IGF-I, IGF-II and its receptor (IGF-IR) are frequently overexpressed in many types of tumors including colorectal cancer (26–29). Overexpression of *IGF-II* mRNA has been observed in colorectal cancer tumors compared with normal tissue (30).

Studies investigating expression of the insulin- and IGF receptors in colon cancer cells during the different stages of cancer, as well as direct effects of glucose, IGFs and insulin on colon cancer cells may help to better understand the role of the insulin-IGF pathway in the pathogenesis of colon cancer. Therefore, in the present study we investigated the potential effects of glucose, insulin and IGFs on proliferation, migration, cell cycle progression and gene expression in colorectal cancer cells originating from four different Dukes’ stages (A, B, C and D).

## Materials and Methods

### Cell Lines and Culture Conditions

SW1116 (ATCC, CCL-233, Lot# 59816766), SW480 (ATCC, CCL-228, Lot# 59932372), SW620, (ATCC, CCL-227, Lot# 61867814) and COLO205 (ATCC, CCL-222, Lot# 618686374) colorectal cancer cell lines were purchased from the American Type Culture Collection (ATCC; Manassas, VA, U.S.A.). The details of each cell line are listed in **Supplementary Table 1**. Initially, all cell lines were cultured in medium recommended by ATCC. According to ATCC, SW1116, SW480 and SW620 were cultured in medium consisting of Leibovitz’s L-15 (Gibco, Ref No: 11414-049) supplemented with 10% FCS (Sigma, F7524, Lot No: 060M3396) and penicillin (Natrium-penicillin G; 1x10^5^ U/liter) in an incubator at 37°C without CO_2_. COLO205 was cultured in medium consisting of RPMI-1640 (Gibco, Ref. No: 11879-020) supplemented with 10% FCS (Sigma, F7524) and penicillin (1x10^5^ U/liter) in an incubator at 5% CO_2_ and 37°C. The cells were first adapted to the medium as recommended by the ATCC for one week, then all the colorectal cancer cell lines were cultured in a common medium to compare all data under the same conditions. For all cell lines, the experimental culture medium consisted of DMEM medium (Gibco, Ref No: 11966-025) supplemented with 10% FCS (Sigma, F7524), penicillin (1x10^5^ U/liter) and glucose (5 mmol/L) in an incubator at 5% CO_2_ and 37°C. Cells were harvested with trypsin solution (0.05%)-EDTA (0.53 mM) (Gibco, Ref. No: 15400-054). Cells were processed through a semi-automated image-based cell analyzer (Cedex XS Innovatis, Roche, The Netherlands), to determine cell concentration and viability based on the Trypan Blue exclusion method.

### Drugs and Reagents

IGF-I (Sigma, I3769) and IGF-II (Sigma, I2526) were purchased from Sigma-Aldrich (Zwijndrecht, The Netherlands). Human recombinant insulin was obtained from Sanofi-Aventis (Insuman Rapid U100). IGF-I and IGF-II were stored at -20°C; insulin was stored at 4°C. Stock solutions of IGF-I and IGF-II were constituted in 0.01 M of acetic acid, insulin was constituted in distilled water, all according to the manufacturer’s instruction.

### Cell Proliferation Assay

For each cell line the optimal cell number plating density in medium containing 5 mmol/L glucose with different fetal calf serum (FCS) concentrations (0.1%, 0.5%, 1%, 10%) was determined. The cells were plated in 24 well plates at increasing cell density (3.125, 6.250, 12.500, 25.000, 50.000, 100.000 cells/mL per well). For each cell line the minimum FCS concentration was determined to study the effects of IGFs and insulin on colorectal cancer cell proliferation. Except for cell line SW620, the growth of the other three cell lines was relatively FCS independent and grew even in medium containing very low FCS (0.1%). To compare all data under equal conditions 0.5% FCS was selected as plating FCS concentration. After determination of optimal minimum FCS concentration for growth of cell lines, the optimal cell number density was determined after 3 days for each cell line in medium with 0.5% FCS (this was for SW1116: 200.000 cells/mL; SW620: 50.000 cells/mL; SW480: 50.000 cell/mL; COLO205: 80.000 cells/mL) and after 7 days (this was for SW1116: 100.000 cells/mL; SW620: 12.500 cells/mL; SW480: 12.500 cell/mL; COLO205: 40.000 cells/mL) (data not shown).

After trypsinization, the cells were plated in 1 mL of medium in 24 well plates at previously obtained optimal cell density. Plates were placed in a 37°C, 5% CO_2_ incubator and cells were allowed to attach overnight. The next day, after washing the plates two times, the culture medium was replaced with 1 mL/well medium containing 0.5% FCS with 5 mmol/L, 12.5 mmol/L or 25 mmol/L glucose to evaluate the effect of different glucose concentration on colorectal cancer cell proliferation. For experiments testing the effects of IGFs and insulin, cells were allowed to attach for one day in medium with 0.5% FCS, washed twice as described above, after which the culture medium was replaced with 1 mL/well medium containing 0.5% FCS, 5 mmol/L or 25 mmol/L glucose and increasing concentrations of IGF-I, IGF-II or insulin (0.01 nmol/L, 0.1 nmol/L, 1 nmol/L, 5 nmol/L, 10 nmol/L). After 3 and 7 days (medium and compounds refreshed at day 3), the cells were harvested for DNA measurement. Measurement of total DNA contents was performed using the bisbenzimide fluorescent dye (Hoechst 33258; Sigma, #B2883) (Boehring Diagnostics, La Jolla, CA) as previously reported (31). The experiments were repeated twice, and each experiment was performed in quadruplicate.

### Cell Cycle Assay

For cell cycle analysis, the colorectal cancer cell lines SW620 and COLO205 were treated with 10 nmol/L IGF-I, IGF-II or insulin. After 3 days, cells were harvested, washed with NaCl, fixed with ice-cold 70% EtOH, and stored at -20°C until analysis. Analyses were performed using the Muse cell cycle assay kit using a Muse cell analyzer (Merck Millipore) according to the manufacturer’s instructions. The experiments were repeated twice, and each experiment was performed in triplicate.

### Cell Migration Assay

The *in vitro* cell migration was measured by the scratch assay method as previously described (32), with some modifications. After trypsinization, cells were plated in 2 mL of medium containing 10% FCS, 5 mmol/L or 25 mmol/L glucose in poly-lysine (10 µg/mL) coated 12-wells plates and placed in a 37°C, 5% CO_2_ incubator. Cells were grown until a confluent monolayer was formed. With a 200 μL pipet tip a scratch was made in the cell monolayer. The debris was removed by washing the cells with 1 mL of growth medium containing 0.5% FCS, 5 mmol/L or 25 mmol/L glucose. Thereafter, 2 mL of medium containing 0.5% FCS, 5 mmol/L or 25 mmol/L glucose and the different compounds of interest (IGF-I, IGF-II and insulin at concentration 10 nmol/L) were added in triplicate. The ability of cells to migrate into the scratch area (wound coverage) was assessed after 2, 4 and 8-hours by comparing the 0- and 2, 4 and 8-hour photomicrographs (Zeiss, Axiovert 40c, x50 magnification) of 4 fixed points along the scratch area. The percentage of non-recovered scratch area was calculated by dividing the non-recovered area after 2, 4 and 8-hours by the initial scratch area (t=0) using image software (https://imagej.nih.gov/ij/). All the experiments were repeated twice. Unfortunately, SW1116 cells did not form a solid attached monolayer and, therefore, it was not possible to perform migration experiments for these cells using the scratch assay method.

### Total RNA Isolation and Quantitative RT-PCR

Total RNA was isolated using a commercially available kit (High Pure RNA kit; Roche; Cat. No: 11828665001). The cDNA synthesis has been described previously (33). In brief, cDNA was synthesized using 500 ng total RNA in RT cocktail. The RT Cocktail consisting of super Reverse Transcriptase (RT) buffer (HT Biotechnology Ltd., Cambridge, UK), 2 U/µL super RT (HT Biotechnology), 20 nmol of each deoxynucleotide triphosphate (HT Biotechnology), 0.005 µg/µL oligo dTprimer (Invitrogen), 10 U/µL RNAse inhibitor (HT Biotechnology), 1 µL ddH_2_O in a final volume of 20 µL. After 1h incubation at 40°C, cDNA was diluted five times. The resulting cDNA was analyzed immediately by real-time PCR or stored at +4°C for further use.

The expression of mRNA of the IGF system (I*GF-I, IGF-II, IGF-IR, IGF-IIR, IRA, IRB, IGFBP-1, IGFBP-3, IGFBP-5* and *IGFBP-6*) in human colorectal cancer cells was evaluated by quantitative RT-PCR as previously described (33). In brief, quantitative PCR was performed by TaqMan Gold nuclease assay (Perkin Elmer Corporation, Foster City, CA, USA) and the ABI-PRISM-7900 sequence Detection System (Perkin Elmer, Groningen, The Netherlands) for real-time amplifications, according to the manufacturer’s protocol. PCR conditions were 95°C for 10 min, followed by 45 cycles of 95°C for 15s and 60°C for 1 min. Hypoxanthine-guanine phosphoribosyl transferase-1 (*HPRT1*; Sigma-Aldrich), glucuronidase beta (*GUSB*; Thermo Fisher Scientific) and beta-actin (*ACTB*; Thermo Fisher Scientific) were used to normalize mRNA levels. PCR efficiencies (E) were calculated for the primer–probe combinations used (**Supplementary Table 2**) and the relative expression of genes was calculated using the comparative threshold method, 2^-ΔCt^. For accurate RT-qPCR expression profiling of the colorectal cancer cell lines, *HPRT, GUSB*, and *ACTB* were determined to normalize mRNA levels using the method as previously described (34). The sequence of the used primers and probes are listed in **Supplementary Table 2**. Ct values >35 did not yield consistent results and were considered below the detection limit of the assay.

### Statistical Analysis

All the experiments were carried out at least twice. Repeated experiments gave comparable results. For the statistical analysis statistical software GraphPad Prisim 6.0 (GraphPad software, San Diego, CA) was used. Comparative statistical evaluation among groups was performed by a one- -way ANOVA test. When statistically significant differences were found, a comparison between groups was made using the Newman-Keuls test. In all analysis, a value of p<0.05 was considered statistically significant. Data are reported as mean ± SEM.

## Results

### Effects of Glucose on Colorectal Cancer Cell Proliferation

**Figure 1:**
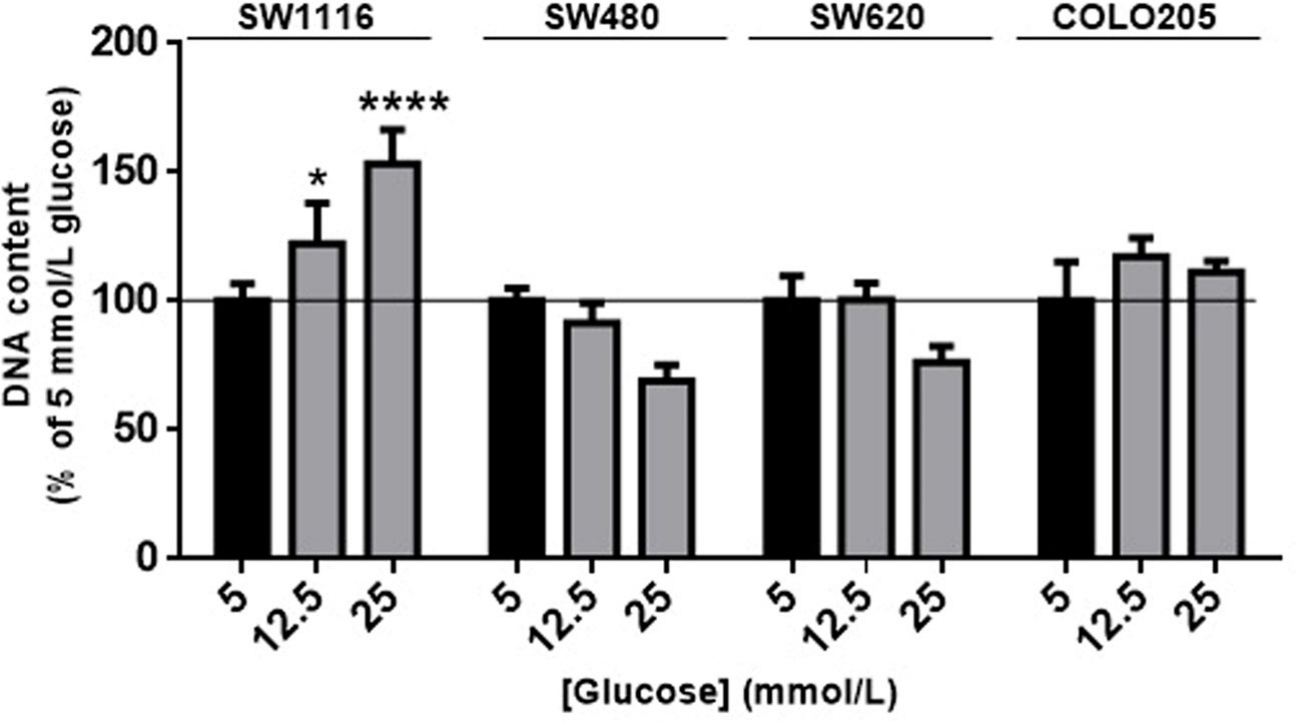
The effect of glucose on cell proliferation after 7 days. SW1116, SW480, SW620 and COLO205 cells were incubated for 7 days with increasing concentrations of glucose (5 - 25 mmol/L). Results are expressed as DNA content/well and as the percentage of the DNA content of 5 mmol/L glucose (black bars). Values represent the mean ± SEM of at least two independent experiments in quadruplicate. *p < 0.05, ****p < 0.0001 *versus* control.

First, we evaluated the effects of increasing concentrations of glucose (5 mmol/L, 12.5 mmol/L, 25 mmol/L) on colorectal cancer cell proliferation (**Figure 1**). Glucose (5 mmol/L) was considered to mimic physiological conditions and used as control (black bars in **Figure 1**). After 7 days of incubation, cell number of SW1116 (Dukes A) cells (reflected by total DNA content) progressively increased with increasing glucose concentrations, and a maximal increase in cell growth of 60% was observed at 25 mmol/L glucose (**Figure 1**). For SW480 cells (Dukes B), SW620 cells (Dukes C) and COLO205 cells (Dukes D), no statistically significant differences in proliferation were observed at all tested glucose concentrations.

### Effects of IGF-I, IGF-II, and Insulin on Colorectal Cancer Cell Proliferation

**Figure 2:**
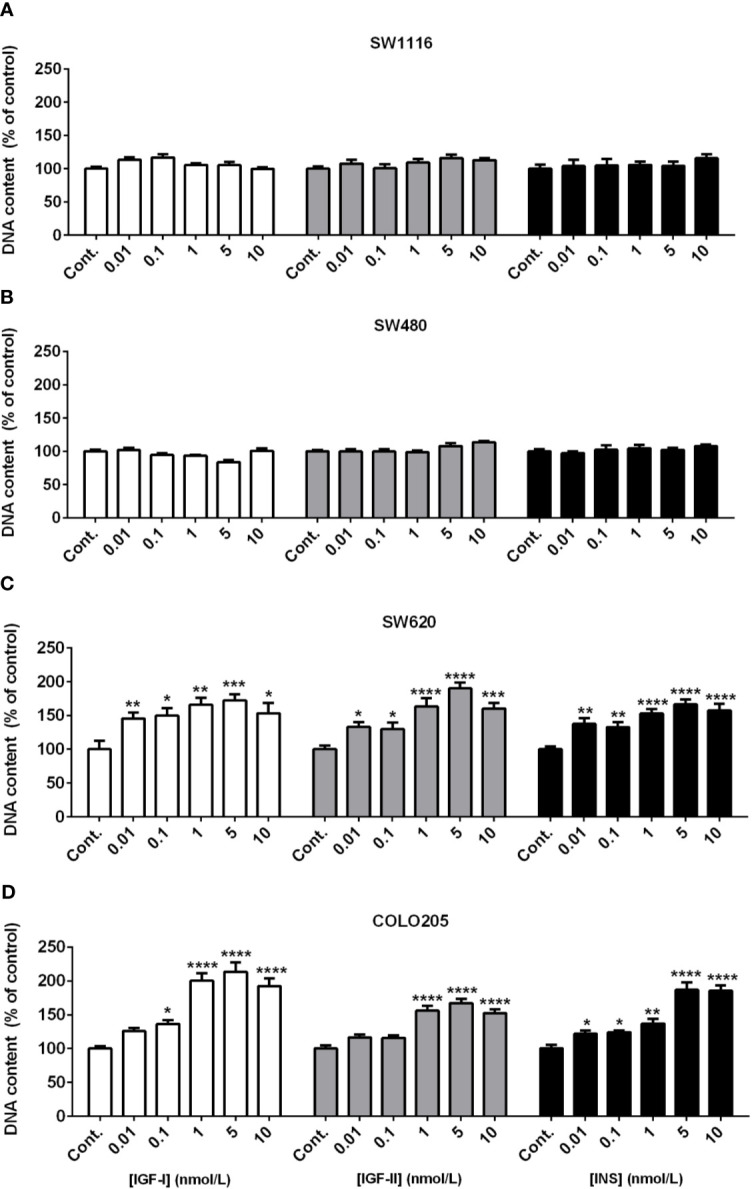
Dose dependent effect of 7-days treatment with increasing concentrations (range 0.01-10 nmol/L) of IGF-I (open bars), IGF-II (grey bars) or insulin (black bars) on SW1116 **(A)**, SW480 **(B)**, SW620 **(C)** and COLO205 **(D)** cell proliferation in 5 mmol/L glucose. Results are expressed as DNA content/well and as the percentage of untreated control. Values represent the mean ± SEM of at least two independent experiments in quadruplicate. *p < 0.05, **p < 0.01, ***p < 0.001, ****p < 0.0001 *versus* control.

When SW116 cells (Dukes A) and SW480 (Dukes B) were stimulated with IGF-I, IGF-II and insulin no stimulatory effects on cell growth were observed both at 5 mmol/L (**Figures 2A, B**) and 25 mmol/L glucose (**Supplementary Figures 1A, B**). On the other hand, IGF-I, IGF-II and insulin significantly stimulated cell growth of SW620 (Dukes C) and COLO205 cells (Dukes D) in a dose-dependent manner, both at 5 and 25 mmol/L glucose (**Figures 2C, D** and **Supplementary Figures 1C, D**). At 5 mmol/L glucose, IGF-I and insulin already significantly stimulated cell growth at the lowest doses tested (0.01-0.1 nM), whereas IGF-II stimulated cell growth at slightly higher concentrations (≥ 1 nmol/L; **Figure 2**, lower panels). At 25 mmol/L a slightly different pattern of cell growth was observed in SW620 cells (Dukes C), and stimulation by IGF-II appeared more potent compared to IGF-I (**Supplementary Figure 1**).

### Effects of IGFs and Insulin on Colorectal Cancer Cell Cycle Progression

**Figure 3:**
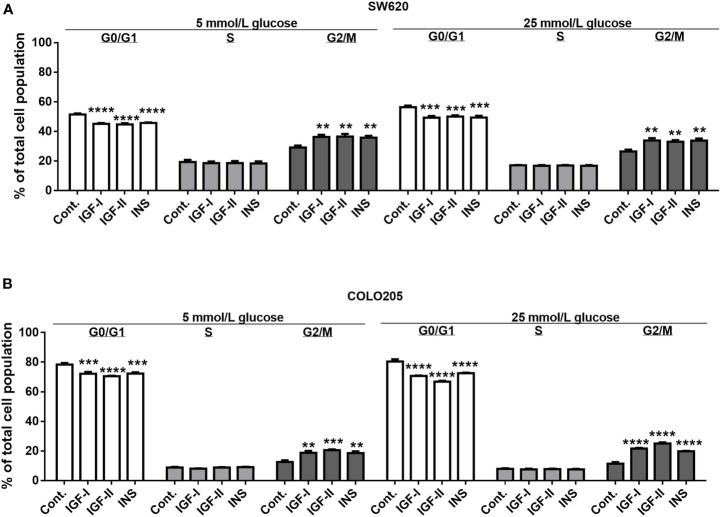
Effect of 3-days treatment with the growth factors IGF-I, IGF-II and insulin (INS) on cell cycle distribution in SW620 **(A)** and COLO205 **(B)** cell lines. Two different conditions were tested: medium containing 0.5% FCS with 5 mmol/L glucose (5 mmol/L) or 0.5% FCS with 25 mmol/L glucose (25 mmol/L). The cells were incubated for 3 days with 10 nmol/L IGF-I, IGF-II or insulin, respectively. Values are expressed as the percentage of untreated control and represent the mean ± SEM of at least two independent experiments in triplicate. **p < 0.01, ***p < 0.001, ****p < 0.0001 *versus* control.

As SW620 (Dukes C) and COLO205 cells (Dukes D) were the only two cell lines that in our hands were stimulated by IGFs and insulin, we also analyzed the effects of these compounds on cell cycle progression (**Figure 3**). The percentage of SW620 cells in G2/M phase significantly increased b Both at 5 and 25 mmol/L glucose, when stimulated by 10 nmol/L IGF-I (to 36.3%, p<0.001 and 33.8%, p<0.0001, respectively), 10 nmol/L IGF-II (to 36.6%, p<0.001 and 33%, p<0.0001, respectively) and 10 nmol/L insulin (to 35.8%, p<0.001 and 33.8%, p<0.0001, respectively), compared to control (29.2%, 26.5%) (**Figure 3A**). A similar change in G2/M phase was observed in COLO205 cells after 10 nmol/L stimulation with IGF-I, IGF-II or insulin. There was an increase in G2/M of to 18.8%, 20.6% and 18.6%, respectively (p<0.0001), compared to control (12.7%) in 5 mmol/L glucose, and to 21.7%, 25.2% and 19.9%, respectively (p<0.0001), compared to control (11.5%) in 25 mmol/L glucose (**Figure 3B**).

In both SW620 cells and COLO205 cells, the G2/M phase accumulation was accompanied by a statistically significant decrease of cells in the G0/G1 phase (**Figures 3A, B**).

### Basal and Growth Factor Stimulated Migration

**Figure 4:**
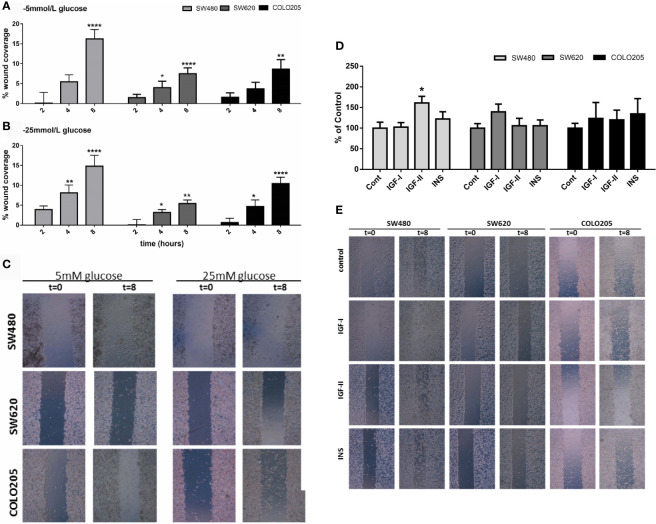
Panel **(A)** and **(B)**: Cell migration of SW480 (light grey bars), SW620 (grey bars) and COLO205 (black bars) cells at 2, 4, 8 hours after scratch in medium containing 5 mmol/L glucose **(A)** or 25 mmol/L glucose **(B)**. The percentage of non-recovered wound area was calculated by dividing the non-recovered area after 8 hours by the initial wound area at time 0 hours. **(C)** Representative pictures of the scratch at 0 time and 8 hours after scratch in SW480, SW620 and COLO205 cells in medium containing 5 mmol/L glucose (left panel) or 25 mmol/L glucose (right panel). Original magnification is x50. **(D)** Percentage of cell migration of SW480, SW620 and COLO205 cells after 8 hours of incubation with 10 nmol/L IGF-I, IGF-II or insulin, respectively, in medium containing 5 mmol/L glucose. **(E)** Representative pictures of the scratch of SW480, SW620 and COLO205 cells at t = 0 and t = 8 hours of incubation with 10 nmol/L IGF-I, IGF-II or insulin, respectively, in medium containing 5 mmol/L glucose. Original magnification x50. Values are expressed as the percentage of wound closure compared to t=0 and represent the mean ± SEM of at least two independent experiments performed in triplicate. *p < 0.05, **p < 0.01, ****p < 0.0001 *versus* control.

To assess cell migration, a scratch wound healing assay method was used. Basal cell migration was studied in culture medium containing 5 mmol/L glucose (**Figure 4A**) and 25 mmol/L glucose (**Figure 4B**). Unfortunately, due to their low attachment, it was impossible to measure migration in SW1116 cells (Dukes A). All three remaining cell lines showed a relative low migration rate and SW480 cells (Dukes B) displayed a higher migration rate compared to SW620 (Dukes C) and COLO205 cells (Dukes D) (**Figures 4A–C**). When cells were cultured in 5 mmol/L glucose and 25 mmol/L glucose, the percentage of wound closure after 8 hours was 16.25% and 14.82% for the SW480 cells; 7.49% and 5.45% for SW620 cells; and 8.66% and 10.43% for COLO205 cells, respectively.

Furthermore, we only observed in SW480 cells a significant increase in migration after 10 nmol/L IGF-II in 5, but not in 25 mmol/L glucose (**Figures 4D, E** and **Supplementary Figure 2**, respectively). No statistically significant stimulatory effects on migration were observed when SW620 and COLO205 cells were cultured in 5 or 25 mmol/L glucose (**Supplementary Figure 2**).

### mRNA Expression of Components of the IGF Pathway in Human Colorectal Cancer Cells

**Figure 5:**
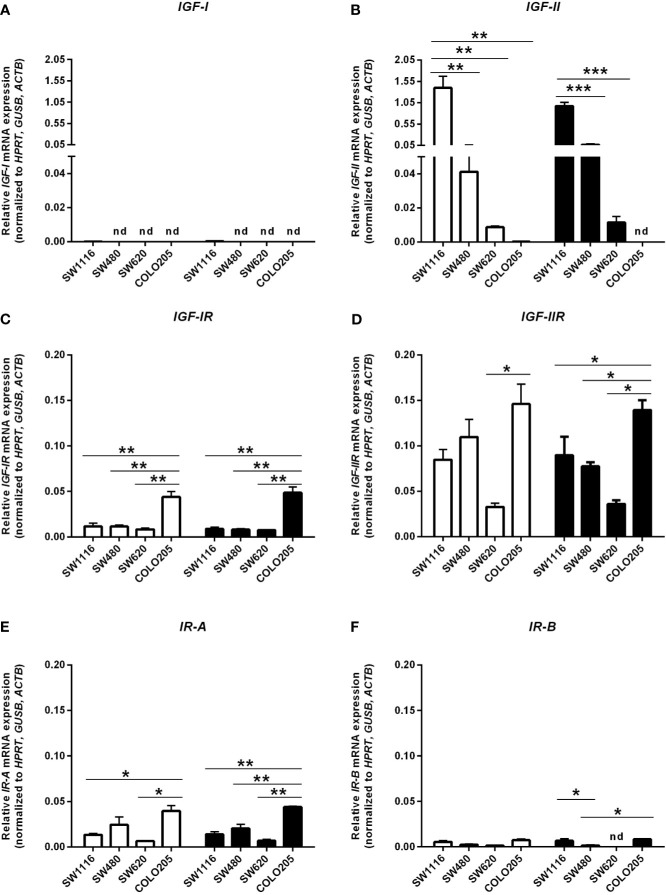
mRNA expression levels of the *IGF-I* **(A)**, *IGF-II* **(B)**, *IGF-IR* **(C)**, *IGF-IIR* **(D)**, *>IR-A* **(E)** and *IR-B* **(F)** (expressed as relative mRNA expression, normalized to the house-keeping genes *HPRT*, *GUSB* and *ACTB*) in four human colorectal cancer cell lines. Two different conditions were tested: medium containing 5 mmol/L glucose (open bars) or 25 mmol/L glucose (black bars). Values represent the mean ± SEM of two independent experiments. *p < 0.05, **p < 0.01, ***p < 0.001. nd, not detectable.

**Figure 6:**
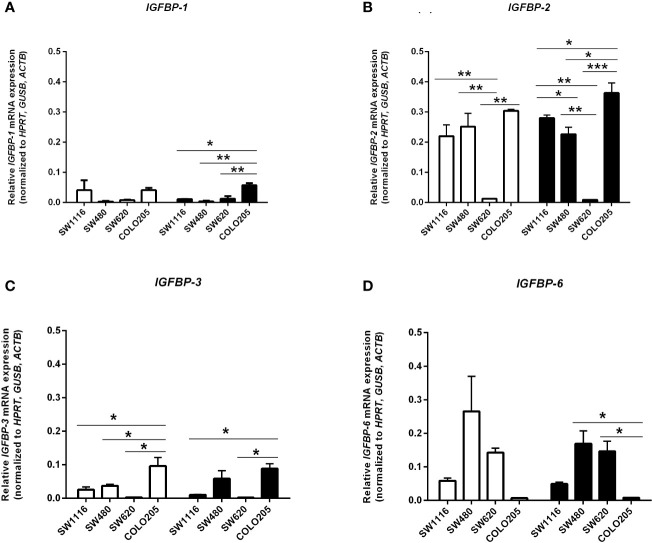
mRNA expression levels of the *IGFBP-1* **(A)**, *IGFBP-2* **(B)**, *IGFBP-3* **(C)** and *IGFBP-6* **(D)** (expressed as relative mRNA expression, normalized to the house-keeping genes *HPRT, GUSB* and *ACTB*) in four human colorectal cancer cell lines. Two different conditions were tested: medium containing 5 mmol/L glucose (open bars) or 25 mmol/L glucose (black bars). Values represent the mean ± SEM of two independent experiments. *p < 0.05, **p < 0.01, ***p < 0.001.

In cells cultured in medium containing 5 mmol/L or 25 mmol/L glucose mRNA expression of *IGF-I* and *IGF-II* (**Figures 5A, B**), mRNA expression of the *IGF-IR, IGF-IIR, IR-A* and *IR-B* (**Figures 5C–F**) and mRNA expression of *IGFBP-1, IGFBP-2, IGFBP-3* and *IGFBP-6* (**Figures 6A–D**) were measured by quantitative RT-PCR. There were no major differences in mRNA expression of *IGF-I, IGF-II, IGF-IR, IGF-IIR, IR-A* or *IR-B* between cells cultured in 5 or 25 mmol/L glucose. Expression of IGF-I mRNA was very low in SW1116 cells (Dukes A) and not detectable in SW480 (Dukes B), SW620 (Dukes C) and COLO205 (Dukes D) (**Figure 5A**). Expression of IGF-II mRNA showed a progressive decrease from cells derived from Dukes’ stage A and B tumors (SW1116 and SW480) towards cells from Dukes’ stage C and D tumors (SW620 and COLO205) (**Figure 5B**). Moreover, SW620 cells had the lowest expression levels of *IGF-IIR, IR-A, IR-B*, compared to the other cell lines (**Figures 5D–F**). In COLO205 cells, mRNA expression of the *IGF-IR, IGF-IIR*, as well as *IR-A* and *IR-B* was relatively high, whereas the mRNA expression of *IGF-I* and *IGF-II* was undetectable and very low, respectively (**Figures 5A–F**). The ratio *IGF-IR/IGF-IIR* progressively increased from cells derived from Dukes’ stage A and B tumors to cells derived from Dukes’ stage C and D tumors. The *IR-A/IR-B* ratio was lowest in SW1116 (Dukes’ stage A) and highest in SW480 (Dukes’ stage B) tumor cells (at 5 mmol/L glucose; **Table 1**; at 25 mmol/L glucose; **Supplementary Table 3**). *IGFBP-2* and *IGFBP-3* expression in COLO205 cells was relatively high compared to the three other studied cell lines, whereas SW620 cells had very low *IGFBP-2* and *IGFBP-3* expression (**Figure 6**).

**Table 1:** Summarizing table of all data of cells in 5 mmol/L glucose condition.

Column1	SW1116	SW480	SW620	COLO205
**Duke stage**	A	B	C	D
**Glucose dependency**	yes	no	no	no
**IGF-I proliferation**	no	no	yes	yes
**IGF-II proliferation**	no	no	yes	yes
**INS proliferation**	no	no	yes	yes
**Relative mRNA expression**				
**IGF-I**	0,00013 ± 0,0001	N.D.	N.D.	N.D.
**IGF-II**	1,397 ± 0,271	0,041 ± 0,018	0,009 ± 0,001	0,0003 ± 0,0003
**IG-IR**	0,012 ± 0,003	0,012 ± 0,001	0,008 ± 0,001	0,044 ± 0,006
**IGF-IIR**	0,085 ± 0,011	0,110 ± 0,019	0,033 ± 0,004	0,146 ± 0,022
**Ratio IGF-IR/IGF-IIR**	0,137	0,105	0,256	0,301
**IR-A**	0,013 ± 0,002	0,025 ± 0,008	0,007 ± 0,001	0,040 ± 0,006
**IR-B**	0,005 ± 0,002	0,002 ± 0,001	0,001 ± 0,0003	0,007 ± 0,001
**Ratio IR-A/IR-B**	2,460	10,686	5,180	5,435
**IGFBP-1**	0,040 ± 0,033	0,003 ± 0,002	0,008 ± 0,001	0,041 ± 0,008
**IGFBP-2**	0,220 ± 0,037	0,251 ± 0,044	0,013 ± 0,001	0,304 ± 0,005
**IGFBP-3**	0,026 ± 0,009	0,037 ± 0,004	0,002 ± 0,0005	0,096 ± 0,025
**IGFBP-6**	0,058 ± 0,008	0,265 ± 0,105	0,142 ± 0,014	0,007 ± 0,001

N.D., not detectable.

## Discussion

To the best of our knowledge, there are no previous studies which systematically have studied 1] whether cell proliferation, cell cycle progression and migration in cells from different stages of colon cancer are modified by glucose and 2] whether effects of IGF-I, IGF-II and insulin on cell proliferation, cell cycle progression and migration change during different stages of colon cancer.

In this study we investigated four well-characterized colorectal cancer cell lines originating from Dukes’ stages A to D. We found that proliferation of SW1116 cells, originating from Dukes’ stage A, progressively increased at higher glucose concentrations. In contrast, glucose did not modify growth of the three other studied cell lines, which originated from Dukes’ stages B to D. These results suggest that glucose is an important growth factor in the early stage of colon tumor growth. Although in a meta-analysis it has been found that diabetes mellitus is associated with an increased risk of colorectal cancer, it is still unclear whether glucose has a role in the initiation of colon cancer independent of hyperinsulinemia (5). High glucose may trigger several direct and indirect mechanisms that cooperate to promote cancer cell proliferation. For example, high glucose may favor anabolic metabolism and thereby fuel tumor growth (35). A link between high glucose levels and cancer was already proposed more than 50 years ago by Warburg (36). He suggested that cancer cells use the glycolysis pathway for respiration and cell division rather than oxidative phosphorylation (36). Hyperglycemia may enhance WNT signaling and thereby proliferation (35). In addition, hyperglycemia may also change IGF-IR signaling. Clemmons et al. found that, following exposure to hyperglycemia, cells undergo a signaling switch leading to an entirely different mechanism to activate both the “metabolic” (PI-3 kinase) and “mitogenic” (MAP) pathways of the IGF-IR (37). This signaling switch leads to increased proliferation and migration (37). Although thus the exact mechanism is not clear at the moment, our study suggests that hyperglycemia influences growth in the early stages of colorectal cancer.

In our study stimulating effects of IGF-I, IGF-II and insulin on cell proliferation were only found in the SW620 and COLO205 cell lines. Both cell lines originate from (metastasized) advanced stages of colon cancer (Dukes’ stage C and D). The stimulating effects of IGF-I, IGF-II and insulin on proliferation were not significantly modified by glucose. In contrast, the IGFs and insulin did not influence proliferation of the SW1116 and SW480 cell lines (which originate from Dukes’ stage A and B, respectively).

Our results suggest that IGFs and insulin are especially important for growth of advanced colorectal cancer cells which have metastasized, but they seem not to play an important role in the early growth of colon cancer cells localized and confined to the bowel. In our study we only studied growth properties of our cell lines in monolayer cultures. In the study of growth properties of cancer cell lines, 3D cell cultures might provide additional information compared to monolayer cell cultures. Therefore, it remains to be studied whether similar differential responses are observed when cells are cultured in 3D cell cultures.

We observed that IGF-I, IGF-II and insulin induced a G2/M arrest in both SW620 (Dukes’ stage C) and COLO205 cells (Dukes’ stage D). This suggests that the IGFs and insulin may positively regulate cell-cycle progression and thereby growth of colon cancer cells.

Numerous epidemiologic studies have found increased cancer risk associated with high circulating IGF-I levels, hyperinsulinemia or both (19, 38). Although these associations do not prove causation there are experimental data to support a role of the IGFs and insulin in the development of cancer. Lowering levels of circulating IGF-I in mice has been shown to inhibit the growth of colon cancer xenografts and to reduce metastatic spread to the liver (39).

We are not aware of any previous studies comparing mRNA expression of parameters of the insulin/IGF system in different Dukes’ stages of colon cancer. Expression of IGF-II mRNA progressively decreased with advanced Dukes’ stages while the expression of IGF-IR and IGF-IIR was highest in the most advanced COLO205 cells (Dukes’ stage D). The proliferating response of the cells to insulin/IGFs was very similar in SW620 and COLO205 cells despite the latter cells having much higher expression of IGF-IR and IR-A, the two potentially most important mitogenic receptors. This suggests that growth of these cells is not directly related to mRNA expression of the IGF-IR and the IR-A, but to other factors like the real number of IGF-IR and IR-A receptors at the cellular surface and the affinity of ligand-receptor interactions.

Our results suggest that local (autocrine and paracrine) production of the IGF-II (but not IGF-I) may play a role in promoting (local) tumor growth in the early non-metastasized stages of colon cancer. Our results further suggest that the endocrine IGF/insulin system becomes more important for growth in the late stages of (metastasized) colorectal cancer, suggesting that a difference in response to insulin/IGFs may be a property acquired with metastatic spread of colon cancer cells to secondary sites. Nevertheless, our results are based on cell line models from colon cancer tissue specimens *in vitro*. Since these models do not perfectly mimic *in vivo* conditions, our results should be interpreted with caution. In addition, we only measured mRNAs but not the actual protein levels of the IGF/insulin system.

Previous evidence suggests that insulin may induce mitosis of normal colorectal epithelial cells, possibly by increasing their energetic metabolism (40). There are two insulin receptors formed *in vivo* by alternative splicing: IR-A, which misses exon 11, and IR-B which contains exon 11 (18). Insulin binds both to the IR-A and the IR-B. However, insulin can also bind to the IGF-IR. This occurs with much lower affinity than to the IR.

The IRs may also form hybrids with the IGF-IR (18). IGF-I binds to the IGF-IR, hybrids or IR, but has a much lower affinity for the IR than IGF-IR while IGF-II binds to the IGF-IR, IR-A and the IGF-IR/IR-A hybrid receptor. Stimulation of the IGF-IR and the IGF-IR/IR-A hybrid receptor by IGF-I or IGF-II predominantly induces proliferation (41). Stimulation of the IR-A by insulin or IGF-II also predominantly induces proliferation whereas stimulation of the IR-B by insulin or the IGF-IR/IR-B by IGF-I predominantly results in metabolic signaling. Frasca et al. have reported an IR-A relative abundance in colorectal cancer cells compared to normal colonic epithelial cells, with median values for the IR-A ranging from 68–73% in cancer to 35–43% in normal tissue (41). Furthermore, it has been found that IR-A is the predominant isoform of the IRs in both the undifferentiated intestinal epithelial stem cells and in the rapidly dividing progenitors of the crypt (42).

Recent data have elucidated molecular mechanisms how the IRs may be involved in cancer (43). The IRs and especially the IR-A is overexpressed in several human malignancies. In our hands the mRNA IR-A/IR-B ratio was also higher in advanced Dukes’ stages, but IR-A mRNA expression did not significantly correlate with Dukes’ stages.

We also evaluated *in vitro* cell migration with the scratch assay method. By analyzing the growth factor stimulated migration within 8 hours after scratch in normal and high glucose conditions we minimalized potential effects of cell proliferation on migration. Only stimulating effects on migration were observed for IGF-II on SW480 cells. In addition, we observed in the three studied cell lines no difference between 5 mmol/L and 25 mmol/L glucose on growth factor stimulated migration. These results suggest only a limited effect of the IGF/insulin system on migration in the early stages of colon cancer.

The activities of IGFs may be modulated by the IGFBPs: they may potentiate or inhibit IGFs action, but also mediate IGF-independent biological effects (44, 45). We observed in our study the highest *IGFBP-1*, *IGFBP-2* and *IGFBP-3* mRNA expression in Dukes’ stage D colorectal cancer cells, having the lowest level *IGFBP-6*. Interestingly, a recent study showed *in vitro* a dose-dependent inhibitory role of IGFBP-6 on proliferation, invasion and migration of colorectal cancer cells (46). However, although IGFBPs may modify effects of IGFs, their modifying effects are through a variety of mechanisms, highly cell type specific and dependent on environment. As such, the relative contributions of the evaluated IGFBPs on colorectal cell growth and migration are difficult to disentangle.

There are several limitations to our study. First, we measured only mRNA and not the actual protein levels of the components IGF/insulin system. Since mRNA levels do not necessarily reflect protein levels, further studies on this are required. In addition, in order to better understand the mechanisms underlying the differential responses of the cell lines to glucose and IGF/insulin, it is worthwhile study the expression of glucose transporters and downstream signaling by assessment of the phosphorylation of IR and IGF-1R and their main effectors such as IRS1, AKT and ERK.

In conclusion, our data suggest that there is a dissociation between the effects of glucose, IGF-I, IGF-II and insulin on cell proliferation between early and late stage colorectal cancer cells. Stimulatory effects of glucose appear to be present only in Dukes’ stage A colorectal cancer cells while growth factor-mediated cell proliferative responses seem to be more prominently present in late Dukes’ stage cells. Moreover, our study suggests that in early stage colorectal cancer stringent glucose control may be important for tumor progression, while in advanced stages of colon cancer inhibition of the endocrine actions of the IGFs and insulin are more important to restrain growth of colon cancer cells.

## Data Availability Statement

The original contributions presented in the study are included in the article/**Supplementary Material**, further inquiries can be directed to the corresponding author.

## Author Contributions

SB, JJ, LH, PK, and SO contributed to designing the experiments and analysis of the data. SB, PK, and FD executed and analyzed the experiments. SB, JJ, ND, SO FK, and LH contributed to the writing and critically revised the manuscript. All authors contributed to the article and approved the submitted version.

## Conflict of Interest

The authors declare that the research was conducted in the absence of any commercial or financial relationships that could be construed as a potential conflict of interest.
