# Supplementary figures and images for: Modifying Effects of Glucose and Insulin/Insulin-Like Growth Factors on Colon Cancer Cells
Source: Front Oncol. 2021 Jul 5;11:645732. doi: 10.3389/fonc.2021.645732 (PMC8287530; doi:10.3389/fonc.2021.645732)

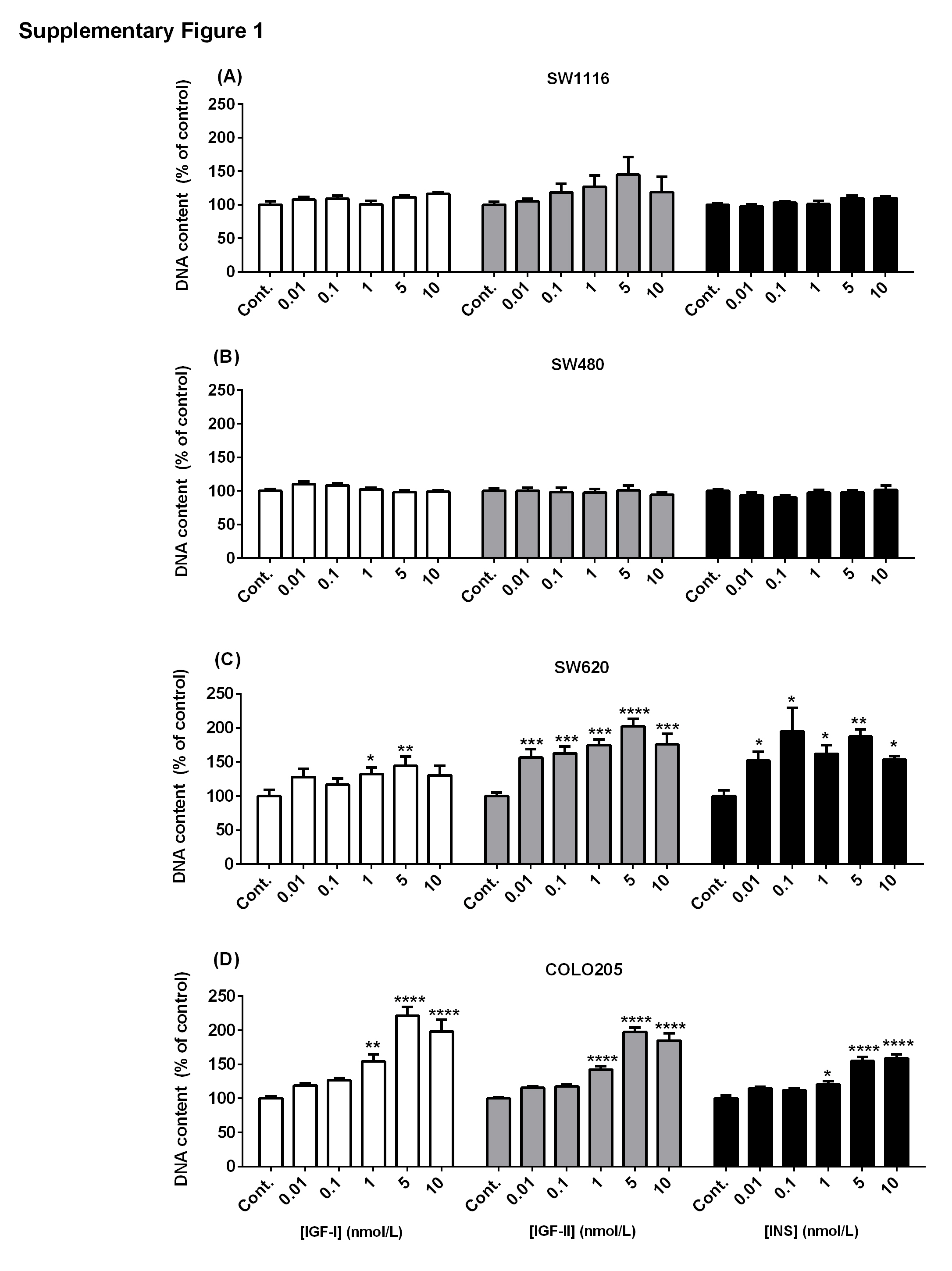

Supplement: Supplementary file 1 [file Image_1.jpeg]
