# Supplementary material for: Modifying Effects of Glucose and Insulin/Insulin-Like Growth Factors on Colon Cancer Cells
Source: Front Oncol. 2021 Jul 5;11:645732. doi: 10.3389/fonc.2021.645732 (PMC8287530; doi:10.3389/fonc.2021.645732)
Supplement: Supplementary file 3 [file Table_1.doc]

**Supplementary Table 1 Colon cancer cell lines origins.**

| **Cell line** | **Tissue** | **Cell type** | **Culture Properties** | **Disease** | **Organism** | **Patient** | **Reference** |
| --- | --- | --- | --- | --- | --- | --- | --- |
| SW1116 | Colon primary | Epithelial | adherent | Dukes' type A,  grade III, colorectal  adenocarcinoma | Homo  sapiens | 73 years  male | 47 |
| SW480 | Colon primary | Epithelial | adherent | Dukes' type B, colorectal  adenocarcinoma | Homo  sapiens | 50 years male | 47 |
| SW620 | Colon Derived from **Metastatic** Site: Lymph Node | Epithelial | adherent | Dukes' type C, colorectal  adenocarcinoma | Homo  sapiens | 51 years male | 47 |
| COLO205 | Colon Derived from **Metastatic** Site: Ascites | Epithelial | mixed, adherent and suspension | Dukes' type D, colorectal  adenocarcinoma | Homo  sapiens | 70 years male | 48 |

1. Leibovitz A, Stinson JC, McCombs WB3rd, McCoy CE, Mazur KC, Mabry ND. Classification of Human Colorectal Adenocarcinoma Cell Lines. Cancer Res (1976) 36:4562.
2. Semple TU, Quinn LA, Woods LK, Moore GE. Tumor and Lymphoid Cell Lines From a Patient With Carcinoma of the Colon for a Cytotoxicity Model. Cancer Res (1978) 38:1345–55.

**Supplementary Table 2 Primers probes and relative amount in PCR mixtures.**

| TARGET GENE | SEQUENCE | Amount (nmol/l) added in the total reaction volume (12.5µl) used for each sample | E factor for primers-probe mixture used |
| --- | --- | --- | --- |
| ***ACTB*** | HU-ACTB 4333762-0710021 applied biosystems:HU-ACTB 20X PROBE DYE FAM-MGB |  | 1.91 |
| ***GUSB*** | HU-GUSB 4333767-1006020 applied biosystems:HU-GUSB 20X PROBE DYE FAM-MGB |  | 2.00 |
| ***HPRT*** |  |  | 1.91 |
|  | Forwad: 5’-CACTGGCAAAACAATGCAGACT-3’ | 500 |  |
|  | Reverse: 5’- GTCTGGCTTATATCCAACACTTCGT-3’ | 500 |  |
|  | Probe: 5’-FAM- CAAGCTTGCGACCTTGACCATCTTTGGA-TAMRA-3’ | 100 |  |
| ***IGF-I*** |  |  | 1.92 |
|  | Forwad: 5’-TTGTGATTTCTTGAAGGTGAAGATG-3’ | 300 |  |
|  | Reverse: 5’-CGTGGCAGAGCTGGTGAAG-3’ | 300 |  |
|  | Probe: 5’-FAM-TACCTGGCGCTGTGCCTGCTCA-TAMRA-3’ | 200 |  |
| ***IGF-II*** |  |  | 1.98 |
|  | Forwad: 5’-CCAAGTCCGAGAGGGACGT-3’ | 300 |  |
|  | Reverse: 5’-TTGGAAGAACTTGCCCACG-3’ | 300 |  |
|  | Probe: 5’-FAM-ACCGTGCTTCCGGACAACTTCCC-TAMRA-3’ | 200 |  |
| ***IGF-IR*** |  |  | 1.85 |
|  | Forward: 5’-CCAAAACTGAAGCCGAGAAG-3’ | 300 |  |
|  | Reverse: 5’- GGGTCGGTGATGTTGTAGGT-3’ | 300 |  |
|  | Probe: 5’-FAM-AAGCAGGAACACCACGGCCG- TAMRA-3’ | 200 |  |
| ***IGF-IIR*** |  |  | 1.87 |
|  | Forward: 5’-ACCGACCCCTCCACGC-3’ | 300 |  |
|  | Reverse: 5’-CCTCCAAGGCCACCTTCAG-3’ | 300 |  |
|  | Probe: 5’-FAM-AGCAGTACGACCTCTCCAGTCTGGCAAA- TAMRA-3’ | 200 |  |
| ***IR-A*** |  |  | 1,86 |
|  | Forward: 5’-CGTTTGAGGATTACCTGCACAA-3’ | 300 |  |
|  | Reverse: 5’-GCCAAGGGACCTGCGTTT-3’ | 300 |  |
|  | Probe: 5’-FAM-TGGTTTTCGTCCCCAGGCCATC- TAMRA-3’ | 100 |  |
| ***IR-B*** |  |  | 1.92 |
|  | Forward: 5’-CCCAGAAAAACCTCTTCAGGC-3’ | 300 |  |
|  | Reverse: 5’-GGACCTGCGTTTCCGAGA-3’ | 300 |  |
|  | Probe: 5’-FAM-CTGGTGCCGAGGACCCTAGGCC- TAMRA-3’ | 200 |  |
| ***IGFBP-1*** |  |  | 1.92 |
|  | Forward: 5’-CACAGGAGACATCAGGAGAAGAAA-3’ | 300 |  |
|  | Reverse: 5’-ACACTGTCTGCTGTGATAAAATCCA-3’ | 300 |  |
|  | Probe: 5’-FAM-TCCAAATTTTACCTGCCAAACTGCAACAA- TAMRA-3’ | 200 |  |
| ***IGFBP-2*** |  |  | 1.88 |
|  | Forward: 5’-GCCCTCTGGAGCACCTCTACT-3’ | 300 |  |
|  | Reverse: 5’-TCTTGCACTGTTTGAGGTTGTACA-3’ | 300 |  |
|  | Probe: 5’-FAM-ACATCCCCAACTGTGACAAGCATGGC- TAMRA-3’ | 200 |  |
| ***IGFBP-3*** |  |  | 1.89 |
|  | Forward: 5’-AGTCCAAGCGGGAGACAGAAT-3’ | 300 |  |
|  | Reverse: 5’-CACATTGAGGAACTTCAGGTGATT-3’ | 300 |  |
|  | Probe: 5’-FAM-TGGTCCCTGCCGTAGAGAAATGGAAGA- TAMRA-3’ | 200 |  |
| ***IGFBP-6*** |  |  | 1.87 |
|  | Forward: 5’-GGCCCATGCCGTAGACATC-3’ | 300 |  |
|  | Reverse: 5’-CTACCGGAAGCGGCAGTGCCG-3’ | 300 |  |
|  | Probe: 5’-FAM-CTACGTGCCCAATTGTGACCATCGAG- TAMRA-3’ | 200 |  |

**Supplementary Table 3** Summarizing table of all data of cells in 25 mmol/L glucose condition.

| **Column1** | **SW1116** | **SW480** | **SW620** | **COLO205** |
| --- | --- | --- | --- | --- |
| **Duke stage** | A | B | C | D |
| **Glucose dependency** | yes | no | no | no |
| **IGF-I proliferation** | no | no | yes | yes |
| **IGF-II proliferation** | no | no | yes | yes |
| **INS proliferation** | no | no | yes | yes |
| **Relative mRNA expression** |  |  |  |  |
| **IGF-I** | 0,0003±0,0001 | N.D. | N.D. | N.D. |
| **IGF-II** | 0,968±0,089 | 0,061±0,022 | 0,011±0,004 | N.D. |
| **IG-IR** | 0,009±0,002 | 0,008±0,001 | 0,008±0,0003 | 0,049±0,006 |
| **IGF-IIR** | 0,09±0,02 | 0,077±0,005 | 0,036±0,004 | 0,139±0,011 |
| **Ratio IGF-IR/IGF-IIR** | 0,101 | 0,105 | 0,214 | 0,349 |
| **IR-A** | 0,014±0,003 | 0,020±0,005 | 0,007±0,002 | 0,044±0,001 |
| **IR-B** | 0,007±0,002 | 0,001±0,001 | N.D. | 0,008±0,001 |
| **Ratio IR-A/IR-B** | 2,117 | 13,851 | N.D. | 5,344 |
| **IGFBP-1** | 0,011±0,0003 | 0,003±0,003 | 0,012±0,009 | 0,057±0,007 |
| **IGFBP-2** | 0,280±0,010 | 0,226±0,023 | 0,009±0,001 | 0,363±0,033 |
| **IGFBP-3** | 0,010±0,001 | 0,059±0,023 | 0,002±0,0002 | 0,088±0,014 |
| **IGFBP-6** | 0,049±0,006 | 0,169±0,038 | 0,146±0,030 | 0,008±0,0004 |

N.D.: not detectable
